# Correlation Between Sonographic Portal Vein Diameter and Flow Velocity With the Clinical Scoring Systems MELD and CTP in Cirrhotic Patients: Is There a Relationship?

**DOI:** 10.4021/gr369w

**Published:** 2012-05-20

**Authors:** Kamran Shateri, Afshin Mohammadi, Farzad Moloudi, Emad Nosair, Mohammad Ghasemi-Rad

**Affiliations:** a Department of Gastroenterology, Urmia University of Medical Sciences, Urmia, Iran; b Department of Radiology, Urmia University of Medical Sciences, Urmia, Iran; c Student Research Committee, Urmia University of Medical Sciences, Urmia, Iran; d Sharjah University, UAE; and Ain Shams University, Egypt; e Genius and Talented Student Organization, Student Research Committee, Urmia University of Medical Sciences, Urmia, Iran

**Keywords:** Sonography, Cirrhosis, Portal vein, Portal vein diameter, Portal vein flow

## Abstract

**Background:**

Liver cirrhosis is defined as a chronic disease of the liver with destruction of the hepatic parenchymal cells. The aim of the current study was to investigate the correlation between sonographic portal vein diameter (PVD) as well as portal flow velocity (PFV) with the clinical scoring systems; CTP and MELD in cirrhotic patients.

**Methods:**

In this cross sectional study, convenience sampling enrolled 108 patients, diagnosed with liver cirrhosis. Blood samples were taken and all patients subsequently underwent Doppler sonography to determine mean portal vein velocity and diameter.

**Results:**

All 108 patients (66 males and 42 females) were enrolled in study. The mean age (± SD) was 50.9 ± 17.6 years (range 13 - 85). The results of the present work revealed weak +ve correlation between MELD and CHILD scores (r = 0.629; P = 0.01). Moreover, the mean PVD showed a little or no +ve correlation with both MELD and CHILD scores (r = 0.216, P = 0.05) and (r = 0.241, P = 0.05) respectively. However, the mean PFV showed no statistical significant relationship with MELD score (P = 0.41).

**Conclusion:**

Sonographic portal vein parameters cannot be a substitute for clinical grading and staging of cirrhosis; and we cannot propose it as a single acceptable diagnostic indicator in grading liver cirrhosis with accuracy.

## Introduction

Liver cirrhosis is defined as a chronic disease of the liver with destruction of the hepatic parenchymal cells. Pathologically, it is characterized by hepatic parenchymal necrosis, and fibrosis of the perivascular connective tissue. There is degeneration of the hepatocyte and formation of irregular regenerating nodules. Clinically, fibrosis and distortion of the portal/periportal architecture causes portal hypertension with the resulting ascites, variceal hemorrhage and hypersplenism [1-3]. The causes of hepatic cirrhosis are multiple, and include congenital, metabolic, inflammatory, and toxic liver diseases; which all end in irreversible destruction of hepatocytes [4]. Cirrhosis is diagnosed histopathologically by presence of fibrosis and regenerative nodules; and clinically by observing stigmata of cirrhosis, ascites and splenomegally. Laboratory findings such as liver enzymes, low serum albumin and increased prothrombin time, as well as endoscopic findings are also essential for diagnosis. However, all these findings are only useful in late stages and have low sensitivity.

Sonography is one of the diagnostic methods used for studying hepatobiliary pathologies, where patients are not exposed to ionizing radiation. It is cheap and easily available, that is why is frequently the first examination performed when liver cirrhosis or portal hypertension is suspected [5], and with the progress of this field it can even be used in staging of cirrhosis and its complications [6-8]. Liver vascular indices calibrated by Sonography are another enhancement in this field. Although it has not been widely used, there are however studies underway [9-11]. Color Doppler sonography can provide valuable measurements of liver vascular indices, and there are data suggesting the validity of using these sonographic indices in grading of liver cirrhosis.

There are differences both in pathology and in clinical signs and symptoms among individual patients. Even in the same patient, there are different pathological and clinical characteristics at different stages. Accordingly, the treatment is very specific for each patient at different stages. Therefore, a clear and correct staging system for cirrhosis is required [12]. Child-Turcotte-Pugh (CTP) score was proved to be a valid independent predictor and prognostic factor of survival. Class C in the CTP grading and a Model for End-Stage Liver Disease (MELD) score higher than 15 were strongly correlated with worse survival. Both clinical scores are the most commonly used system; and must be taken into consideration for adequate evaluation and staging of cirrhosis [13, 14].

According to our best knowledge, there are very few studies investigated the relationship between sonographic portal vein diameter (PVD) and portal flow velocity (PFV) with clinical scoring system. Some studies showed positive relationship and proposed sonography as a good diagnostic modality, while others have totally questioned the role of sonography in diagnosis of cirrhosis [15, 16].

So, since there is no strong evidence regarding the role of sonography in cirrhosis, the aim of the current study was to investigate the correlation between sonographic PVD as well as PFV with the clinical scoring systems; CTP and MELD in cirrhotic patients.

## Materials and Methods

### Patients

In this cross sectional study, 108 patients (66 males and 42 females) diagnosed with liver cirrhosis were used by convenience sampling. The mean age (± SD) was 50.9 ± 17.6 years (range 13 - 85). The inclusion criteria for diagnosis of cirrhosis were spleenomegaly, palmar erythema, spider angioma, and based of laboratory evaluation and liver biopsy. Those of hypoalbunemia, polyclonal gammopathy, laboratory findings such as plasma bilirubin, prothrombin time, transaminase levels, abdominal sonographic findings of spleenomegally, collateral veins in liver and spleen hilum, ascites, heterogenic liver echo and liver border irregularity were all defined as cirrhosis. Those with biopsy proven cirrhosis were also included. The study was approved by the university Institutional Review Board and Ethics Committee. An informed written consent was obtained from all participants. The Ethical Committee of University approved the proposal and the informed consent was obtained from all patients.

### Sampling and scanning techniques

Blood sampling was performed for measuring serum bilirubin, creatinine, albumin, prothrombin time (PT) and International Normalized Ratio (INR). Clinical and sonographic judgments were performed to assess hepatic encephalopathy and ascites. Then the sonography was performed in all patients.

### MELD and CTP scores

The MELD equation used to calculate the severity score was as follows: 9.57 x log_e_ (creatinine mg/dL) + 3.78 x log_e_ (bilirubin mg/dL) + 11.2 x log_e_ (INR) + 6.43 (constant for liver disease etiology) [17]. Minimal values are set to 1.0 for calculation purposes. The maximal serum creatinine level considered within the MELD score equation is 4.0 mg/dL. The CTP score is calculated on the basis of serum bilirubin, serum albumin, PT, level of encephalopathy and level of ascites (Table 1).

**Table 1:** CTP Score, Ascites and Encephalopathy in Our Patients

Clinical Characteristics	Data
CTP Score	No. of patients (%)
Class A (5 - 6)	10 (9.3%)
Class B (7 - 9)	58 (53.7%)
Class C (> 9)	40 (37%)
Ascites	No. of patients (%)
No	22 (20.4%)
Mild	34 (31.5%)
Severe	52 (48.1%)
Encephalopathy	No. of patients (%)
No	89 (82.4%)
Mild	19 (17.6%)
Severe	0

### Sonographic methods

All patients were kept fasting overnight prior to the procedure at our institution. The patients were scanned while in a supine position by a subcostal approach pointing posterocephaled or a right intercostals approach pointing medially.

**Figure 1:**
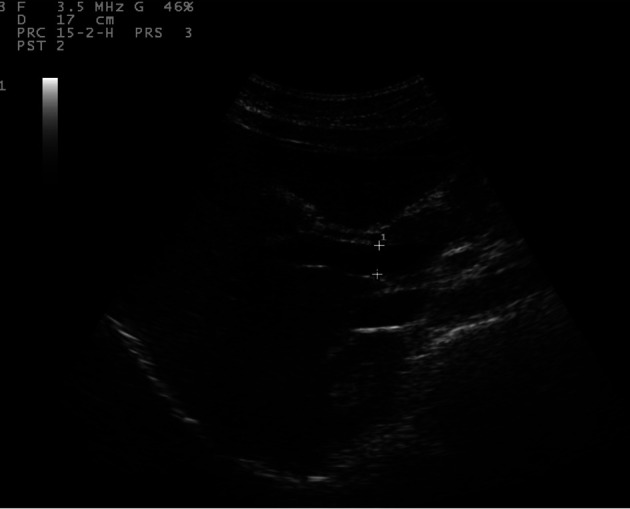
Portal vein diameter (curve arrow) is measured where it crosses anterior to IVC (arrow).

**Figure 2:**
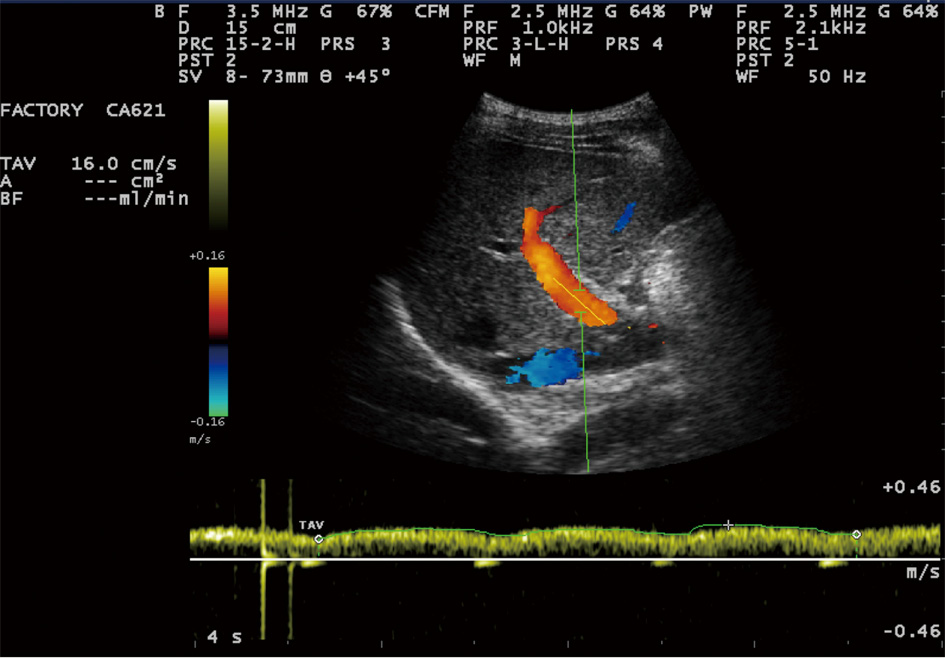
Portal vein time averaging flow velocity (TAV) measurement show: 16cm/s mean velocity of portal vein flow.

**Figure 3:**
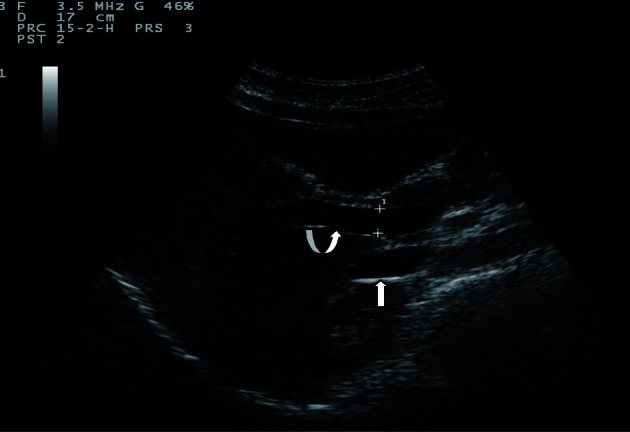
Portal vein peak velocity which is shown to be 21 cm/sec.

Sonographic measurements were done by the same examiner and were repeated for three times to gain the PVD and PFV and were standardized by examining the patients in the supine position and in a state of quite respiration. We measured the diameter of portal vein where the portal vein crosses anterior to the inferior vena cava as (Fig. 1). Color and Duplex Doppler assessment of portal vein flow velocity as time average maximal velocity in cm/s was determined as (Fig. 2, 3). The pulse repetition rate (PRF) was set at minimum to detect flow in patients with portal hypertension and slow flow velocity in portal vein. Doppler angle was less than 60. All examinations were performed using Esaote-mylab 50 US systems (Esaote Biomedical, Genoa, Italy) equipped with a broadband 3.5-5 MHz curvilinear transducer.

### Statistical analysis

We used SPSS version 16.1 for analyzing the data. Chi-square test or Fisher’s exact test (two-tailed) was used for categorical data. Pearson correlation test was used to estimate the strength of the linear correlation (r) and significance between the CHILD and MELD scores; and between CHILD and MELD scores and the corresponding PVD and PFV. P value of < 0.05 was considered significant for each of the mentioned tests.

## Results

In the current study, more than half of the studied patients (53.7%) had score of 7 - 9 (CHILD B), and 37% had score of > 9 (CHILD C); while only 9.3% had score of 5 - 6 (CHILD A). Also, roughly half of the patients (48.1%) developed sever ascites, 31.5% had mild ascites, while 20.4% had no ascites. Regarding encephalopathy, 82.4% of the patients showed no signs of encephalopathy, 17.6% showed signs of mild encephalopathy, while none of the patients had severe encephalopathy (Table 1). The results of the hematology test are shown in (Table 2).

**Table 2:** The Hematological Results of the Total Number of Patients (108)

	Mean ± SD (range)
Serum albumin (g/dL)	3.26 ± 0.83 (1.3 - 6)
Serum bilirubin (mg/dL)	4.61 ± 7.68 (0.2 - 21.1)
Serum creatinine (mg/dL)	1.29 ± 0.95 (0.3 - 5.4)
PT (sec)	16.73 ± 3.94 (11.6 - 34.8)
INR	1.93 ± 1.02 (0.9 - 7.25)

Sonograohic measures shows that, the mean PVD ± SD is 12.11 ± 3.24 mm (range 5 - 20). PVD < 13 mm was observed in 61.1% of the patients; while 38.9% had PVD > 13. Also, 17.6% of the patients showed decreased PFV (< 19 cm/sec), 61.1% had normal range of PFV (19 - 23 cm/sec), while in 21.3% PFV was increased (> 23 cm/sec) (Table 3).

**Table 3:** The Sonographic Measurements of Studied Patients

Sonographic Measures	Data
PVD (mm)	
Mean ± SD (range)	12.11 ± 3.24 (5 - 20)
< 13 (No. of patients - %)	66 (61.1%)
> 13 (No. of patients - %)	42 (38.9%)
PFV (cm/sec)	No. of patients (%)
Normal (19 - 23)	66 (61.1%)
Decreased < 19	19 (17.6%)
Increased > 23	23 (21.3%)
No. of patients with their corresponding (MELD score ± SD)	
Normal (19 - 23)	67 (17.96 ± 9.05)
Decreased < 19	22 (17.97 ± 10.05)
Increased > 23	19 (15.88 ± 7.50)

The results of the present work revealed weak positive correlation between MELD and CHILD scores (r = 0.629; P = 0.01). Moreover, the mean PVD showed a little or no positive correlation with both MELD and CHILD scores (r = 0.216, P = 0.05) and (r = 0.241, P = 0.05) respectively. However, the mean PFV showed no statistical significant relationship with MELD score (P = 0.41) (Table 4).

**Table 4:** No. of Patients (%) of Hematological Results With Their Corresponding CTP Grades

	CTP Grade A	CTP Grade B	CTP Grade C
Serum Albumin (g/dL)	28 (25.9%)	54 (50%)	26 (24.1%)
Serum Bilirubin (mg/dL)	53 (49.1%)	13(12%)	42 (38.9%
INR	59 (54.6%)	25 (23.1%)	24 (22.2%)

## Discussion

Clinical staging of severity of cirrhosis has an important approach in determining the prognosis and early treatment of cirrhotic patients. That is why the MELD and CTP scores are improved and revised. Being a cheap and a non-invasive diagnostic method, Sonography has been valuable in measuring the hepatic hemodynamic changes accompanying cirrhosis and its complications. Thus, in turn it can be of assistance in its clinical grading of severity.

The results of the current study show that the mean PVD ± SD of the cirrhotic patients is 12.11 ± 3.24 mm. This measurement is different from previous studies (< 10 mm) performed to define normal ranges of ultrasound PVD from 6.3 - 9.7 mm [18-21]. Moreover, the mean PVD exhibits a significant relationship with different variables, e.g. body height [19], and respiratory phases [22].

This showed that the PVD has high sensitivity which is reported to be up to 95% [23, 24]. Thus, it is safe to assume that a PVD > 13 mm is a fairly characteristic sign of portal hypertension in the appropriate clinical setting. However, the question is: Is there an associated increase in PVD with the severity of cirrhosis ?”.

The results of this study show little positive correlation between the mean PVD and the severity of cirrhosis. The correlation was significant with both MELD grade (r = 0.216, P = 0.05), and CTP grade (r = 0.241, P = 0.05). The correlation coefficient is about 20% for both. However, it doesn’t show linear relationship. Results from previous studies showed a verity and inconsistency in PVD response towards liver cirrhosis and its hemodynamic changes, and towards its clinical grading.

The results of a study done by Macias et al [25] concluded that the PVD could be used in diagnosis of cirrhosis in sub-clinical and asymptomatic patients, and proposed a PVD cutoff point of 12 mm for diagnosis of cirrhosis. In another study, both sonographic findings of PVD and clinical scoring MELD and CTP showed correlation with liver fibrosis [26].

On the other hand, previous studies documented no significant difference in PVD between cirrhotics and controls [27], or between the compensated and decompensated cirrhosis groups [28], or among various CTP grades suggesting that PVD does not correlate with the high portal pressure and the severity of cirrhosis [15]. Zardi et al [29] reported that the mean PVD slightly but not significantly increased in patients with portal hypertensive gastropathy; and that the oscillatory trend of PVD from control to large size esophageal variances (EV) might indicate that EV may unload portal pressure in the initial phases of portal hypertension; and concluded that PVD was not able to predict EV or large size EV in a large series of patients with cirrhosis.

Other studies reported that the PVD did not positively correlate with the degree of cirrhosis [30], and might not be a reliable indicator of portal hypertension [5, 22]. PVD did not increase with the porto-hepatic venous pressure gradient, or might even decrease with severity of hypertension [31]. Bolondi et al [22] reported that the PVD would decrease with the development of reversed portal vein flow (hepatofugal flow) and/or porto-systemic shunts. These findings coincides with the results of this study, and might explain the little association and absence of linear correlation between PVD with the clinical grades of MELD and CTP in the present study.

In the preset study, weak positive but significant correlation between MELD and CTP was recorded in scoring for cirrhosis (r = 0.629, P = 0.01). This finding is in agreement with previous studies reporting same relationship but with higher correlation coefficient values [32-34].

Duplex sonography represents the best noninvasive technique for assessing PFV in patients with cirrhosis and portal hypertension [35, 36]. Previous studies documented strong correlation between the values of PFV measured by Doppler sonography and MRI in normal subjects [37-39]; however, no correlation of both methods with the portal pressure gradient was found.

The results of our study show no significant relationship between sonographic PFV and the clinical MELD score. This finding is in agreement with Schnider et al [40]. However, many previous studies reported a significant decrease in PFV as the cirrhosis progressed [27], and with increasing CTP grades of severity of cirrhosis [15, 26]; however, the portal blood flow remained normal because of enlarged portal caliber [34]. Also, KoK et al [36] described a reversed portal flow in patients with veno-occlusive disease and portosystemic shunts; and a decrease in the PFV in cirrhotic patients. Moreover, the congestion index of portal vein (CI) shows evidence of higher sensitivity (71%) in detecting cirrhosis than PFV (23%). By using this index, the PFV was significantly reduced, while the cross-sectional area of the portal vein was increased in cirrhotic patients; and despite of portal hypertension, the volume of portal blood flow was well maintained normal [41].

### Conclusion

According to the results of the current study, it seems that, in cirrhosis, the rate of pathologic changes in the portal hemodynamics, as indicated by the sonographic PVD and PFV does not accurately correlate, and does not go in parallel with the rate of progressive deterioration of the heptocellular function, as indicated by the clinical predictors. So, sonographic portal vein parameters cannot be a substitute for clinical grading and staging of cirrhosis; and we cannot propose it as a single acceptable diagnostic indicator in grading liver cirrhosis with accuracy.

## Abbreviations

TAV: time averaging flow velocity
PT: prothrombin time
INR: International Normalized Ratio
CTP: Child-Turcotte-Pugh
MELD: Model for End-Stage Liver Disease
PVD: portal vein diameter
PFV: portal flow velocity.
